# Inflammatory cytokine depletion in severe coronavirus disease 2019 infectious pneumonia: A case report

**DOI:** 10.1097/MD.0000000000023449

**Published:** 2020-12-04

**Authors:** Bo Yang, Jing Yang, Lan Zhou, Cheng Xue, Hongxian Li, Weifeng Hu, Nanmei Liu

**Affiliations:** a Internal Medicine III (Nephrology & Endocrinology), Naval Medical Center of PLA, Second Military Medical University, Shanghai; b The Fourth Department of Infectious Disease, Guanggu Branch of Hubei Province Maternity and Childcare Hospital, Wuhan; c Intensive Care Unit, Naval Medical Center of PLA; d Division of Nephrology, Changzheng Hospital, Second Military Medical University, Shanghai, China.

**Keywords:** case report, convalescent plasma, coronavirus disease 2019, double filtration plasma pheresis, inflammatory cytokine depletion, tocilizumab

## Abstract

**Rationale:**

Multiorgan/system injury was observed in severely infected coronavirus disease 2019 (COVID-19) patients, in addition to viral pneumonia. Recognizing and correcting the key and immediate dysfunctions may reduce mortality.

**Patient concerns:**

A 66-year-old previously healthy male patient was referred to the isolation ward in Guanggu Branch of Hubei Province Maternity and Childcare Hospital with a high fever and nonproductive cough for twenty days.

**Diagnoses:**

Diagnosis of severe COVID-19 infectious pneumonia was established by travel history, clinical features, chest imaging, and a positive oropharyngeal swab specimen result for the severe acute respiratory syndrome coronavirus 2 RT-PCR assay.

**Interventions:**

In addition to standard supportive care, combined inflammatory cytokine depletion therapy (double filtration plasma pheresis and tocilizumab) and convalescent plasma were administered.

**Outcomes:**

The patient's homeostatic parameters (blood pressure, heart rate, spontaneous respiration, SPO2, and blood gas) recovered, along with the recovery on chest imaging. All the intravenous catheters were removed. Supportive care continued for several days, and the patient was transferred to a non-ICU isolation ward.

**Lessons:**

It is not safe to draw causal conclusions between cytokine depletion and clinical manifestation improvement with only 1 case, but this is a potential research direction in facing the COVID-19 crisis.

## 1 Introduction

According to the latest World Health Organization bulletin, the coronavirus disease 2019 (COVID-19) epidemic that began in December 2019 has caused more than 21 million infections and 761,000 deaths worldwide.^[1]^ The disease is caused by a novel coronavirus that was first discovered in Wuhan, China, and has since affected all provinces within the country, and then spread globally. On January 30, the World Health Organization listed the situation as a public health emergency of international concern. COVID-19 is caused by severe acute respiratory syndrome coronavirus 2 (SARS-CoV-2) infection, which is a novel member of the coronavirus family. Multiorgan/system injury has been observed in severely infected patients, in addition to viral pneumonia. Since there is no specific antiviral treatment for COVID-19, supportive care in the ICU plays a crucial role in patient management. Recognizing and correcting the key and immediate dysfunctions may reduce mortality.

We reported a previously healthy 66-year-old man infected with SARS-CoV-2. The disease progressed rapidly, and the patient was transferred to the ICU. Extracorporeal membrane oxygenation (ECMO) was subsequently administered. With the time bought by ECMO and other supportive care, convalescent plasma and multiple cytokine depletion measurements, including double filtration plasma pheresis and tocilizumab, were performed. The patient responded to the treatments, and ECMO was discontinued 1 week later.

## 2 Case presentation

On March 14, 2020, a 66-year-old previously healthy male patient was referred to the isolation ward in Guanggu Branch of Hubei Province Maternity and Childcare Hospital with a high fever and nonproductive cough for twenty days. On March 4, 2020, he visited a local clinic for his symptoms. An oropharyngeal swab specimen was collected and tested positive using the SARS-CoV-2 RT-PCR assay, along with chest CT showing ground-glass opacity in both lungs, which indicated viral pneumonia. Then, he was admitted to the local hospital and given supportive therapy and administered 600 mg methylprednisolone in 10 days. However, his shortness of breath deteriorated while the SPO_2_ decreased to 85% to 89% while on noninvasive ventilation at the time of transfer. On examination, his temperature was 36.4°C, pulse 86/min, respiratory 21/min, and blood pressure 118/64 mm Hg. Supportive care was then provided in the isolation ward, and laboratory tests were also performed (Table 1). On March 15, the SPO_2_ decreased further (80%–85%) with high-flow oxygen therapy (60 L/min). Arterial blood gas analysis showed a pH 7.41, PO_2_ 72 mm Hg, and PCO_2_ 55 mm Hg. Intubation and mechanical ventilation were administered. A 10 cmH_2_O positive end-expiratory pressure and 80% inspired oxygen were applied.

**Table 1:** Lab tests results of the patient at referring.

	Results	Reference range
WBC count	12.4	3.5–9.5 ^∗^10^9/L
Lymphocyte	6.8	20–50%
Monocyte	3.9	3–10%
eosinophil	0.1	0.4–8%
Basophil	0.0	< 1%
neutrophil	89.2	40–75%
RBC count	4.38	4.3–5.8 ^∗^10^12/L
Hb	141	130–175 g/L
Packed cell volume	40.1	40–50%
Mean corpuscular volume	91.7	82–100 fL
Mean corpuscular Hemoglobin concentration	32.1	27–34 pg
Mean corpuscular Hemoglobin	350	316–354 g/L
Red blood cell volume Distribution width	11.8	10.9–15.4%
Platelet count	141	125–350 ^∗^10^9/L
Mean platelet volume	10.9	8–10 fL
Serum total bilirubin	8.0	3.4–20.5 umol/L
Unconjugated bilirubin	4.9	0–14 umol/L
Conjugated bilirubin	3.1	<8.6 umol/L
Serum total protein	61.4	64–83 g/L
Albumin	26.1	35–52 g/L
Globulin	35.3	20–40 g/L
Albumin/globulin	0.74	1–2.4
ALT	53.1	0–55 U/L
AST	36.1	5–34 U/L
ALP	93	40–150 U/L
Gamma-glutamyl transferase	64	12–64 U/L
Bile acid	3.5	<9.67 umol/L
Lactate dehydrogenase	752	125–220 U/L
Blood urea nitrogen	8.9	3.2–7.4 mmol/L
Sodium	139	136–145 mmol/L
Potassium	4.5	3.5–5.1 mmol/L
Chlorine	111	98–108 mmol/L

**Figure 1:**
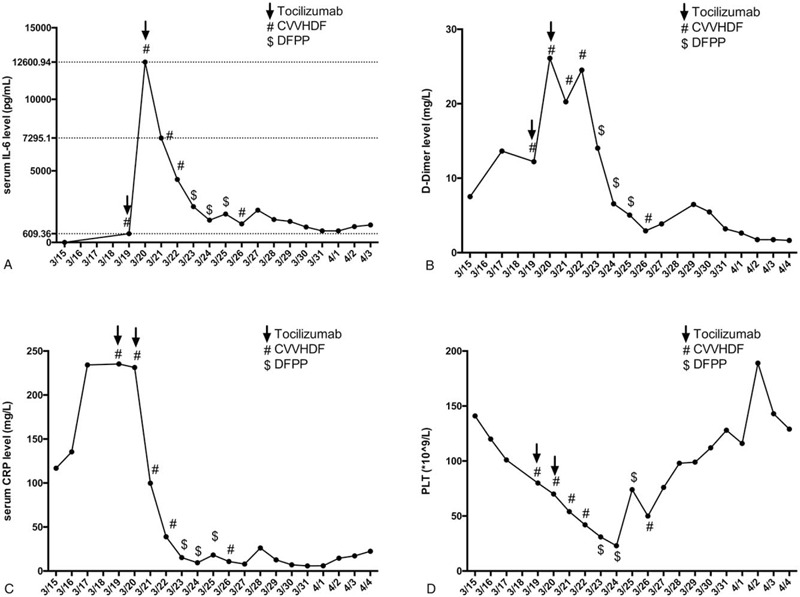
Typical inflammatory markers of the patient: (A) serum IL-6 level (the normal range is <10 pg/mL); (B) D-dimer (the normal range is <0.55 mg/L); (C) serum C-reactive protein (the normal range is <10 mg/L); D) platelet level (the normal range is [125, 350] ^∗^10^9/L).

**Figure 2:**
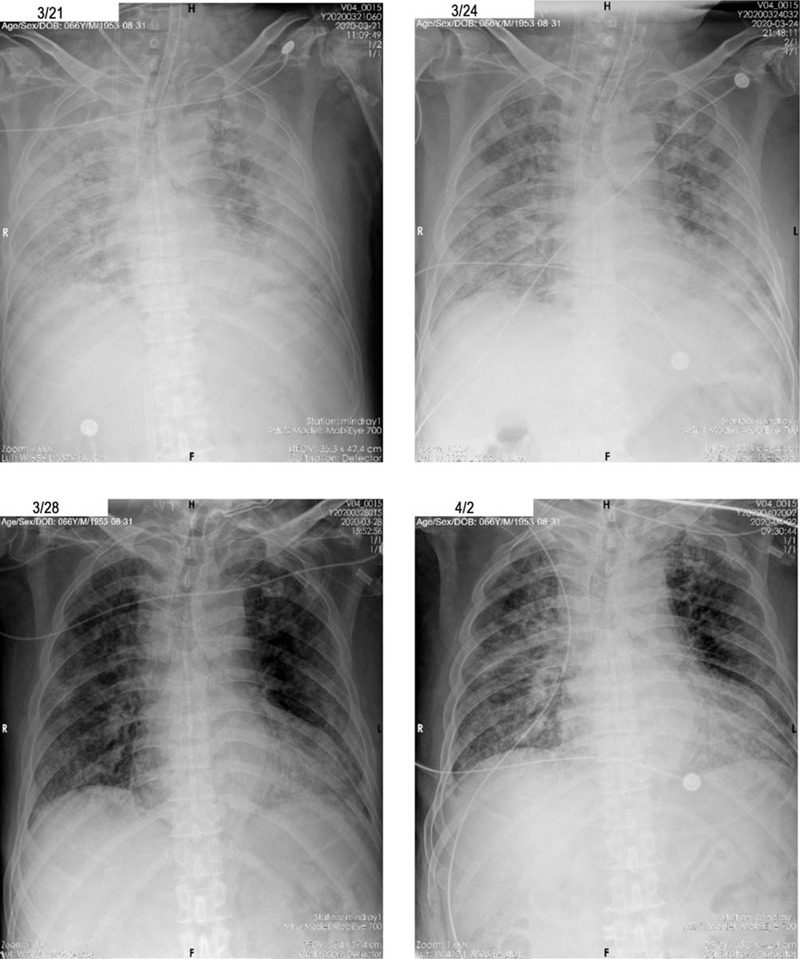
Chest X-ray images of the patient.

The next day, the patient's symptoms of dyspnea and chest tightness deteriorated, and norepinephrine infusion was required to maintain normal blood pressure. Venovenous ECMO was initiated, and the inspired oxygen was downregulated to 40%. After the procedure, his SPO_2_ increased to 98% to 100% and blood pressure 110/70 mm Hg. On March 19, the patient's serum IL-6 was tested, and the result revealed a considerably high IL-6 level. Intravenous tocilizumab (400 mg) was administered; however, the IL-6 level increased even more the following day (Fig. 1 A). Intravenous tocilizumab (400 mg) was repeated. At the same time, the patient also received continuous venous hemodialysis treatment daily from March 19 to March 22 to quickly decrease the IL-6 level. However, the bedside chest X-ray still revealed severe pneumonia, and the clinical manifestations did not improve (Fig. 2). The inflammatory cytokines remained at a high level. The continuous renal replacement therapy (CRRT) protocol was changed to double filtration plasma pheresis (DFPP) with a plasma separator (plasmaflow-08w, Asahi Kasei) and a plasma fractionator (EC-20W, Asahi Kasei) 3 times from March 23 to March 25 before the protocol returned to continuous venous hemodialysis on March 26. The estimated plasma volume was set as 3 L, the substitution fluid was albumin solution, blood flow rate 100 mL/min, plasma flow rate in separator 30 mL/min, and waste flow rate 3.0 mL/min. From March 16 to 25, the patient received multiple blood transfusions (including 400 mL convalescent plasma) to compensate for the volume loss caused by ECMO and CRRT bypass. On 25 March, due to the anticipation of prolonged mechanical ventilation, a tracheotomy was performed. With the time bought by ECMO and CRRT, the patient's homeostasis recovered, along with the recovery of chest imaging. On March 27, ECMO was discontinued according to the ECMO expert evaluation. His SPO_2_ remained at 100% in the condition of mechanical ventilation after ECMO discontinuation. Since the hemodynamics and blood gas improved, all the intravenous catheters were removed on March 28. Supportive care continued for several days, and the patient was transferred to a non-ICU isolation ward. The typical inflammatory markers (including IL-6, D-Dimer, C-reactive protein, and platelet) during this period of time are shown in Figure 1A–D, while the D-dimer (Fig. 1B) and platelet (Fig. 1D) results should be interpreted with caution due to the use of anticoagulants.

## 3 Discussion and conclusions

In most severe COVID-19 patients, acute respiratory distress syndrome is usually the most obvious manifestation and is the leading cause of mortality.^[2]^ In the case of failing to maintain ideal SPO_2_ with mechanical ventilation, ECMO is required. Underlying disease progression could be approached only with stabilized hemodynamics and SPO_2_. Early and sufficient supportive care can buy time for inflammation clearance.

Emerging evidence suggests that cytokine storm syndrome exists in severe COVID-19 patients.^[3]^ It may contribute to the conversion from nonsevere pneumonia to acute respiratory distress syndrome or even MODS. There is also research suggesting monitoring IL-6 to evaluate cytokine storm syndrome in severe cases.^[4]^ In our case, we applied the suggestion and conducted investigational therapy to block IL-6.^[5]^ In addition, multiple measures, including DFPP and tocilizumab, were also used to remove cytokines from the plasma. Within 1 week of intensive care, the patient's condition improved considerably. A previous study has already revealed the potential benefit of using convalescent plasma in COVID-19.^[6]^ The use of tocilizumab in COVID-19 has been tested, but no results have been reported thus far. Although there is no solid evidence supporting the use of DFPP in COVID-19, the rationale for conducting DFPP is also the existence of a cytokine storm and the high level of IL-6. Previous studies verified the effectiveness of DFFP in selectively removing pathogenic substances.^[7–9]^

In summary, it is not safe to draw causal conclusions between cytokine depletion and clinical manifestation improvement with only 1 case, which is a potential research direction in addressing the COVID-19 crisis.

## Author contributions

**Conceptualization:** Nanmei Liu.

**Data curation:** Jing Yang, Lan Zhou, Cheng Xue, Hongxian Li, Weifeng Hu, Nanmei Liu.

**Writing – original draft:** Bo Yang.

**Writing – review & editing:** Bo Yang.
